# Association between fundamental motor skills and executive function in preschool children: A cross-sectional study

**DOI:** 10.3389/fpsyg.2022.978994

**Published:** 2022-08-25

**Authors:** Xiaowei Han, Meiling Zhao, Zhe Kong, Jun Xie

**Affiliations:** ^1^Faculty of Education, Beijing Normal University, Beijing, China; ^2^Graduate School, Capital University of Physical Education and Sports, Beijing, China; ^3^School of Leisure and Social Sports, Capital University of Physical Education and Sports, Beijing, China

**Keywords:** fundamental motor skills, executive function, preschool children, locomotor skills, association

## Abstract

**Objective:**

The main purpose of this study was to explore the association between early fundamental motor skills (FMS) and executive function (EF) in preschool children.

**Methods:**

A total of 394 young children (4.07 ± 0.76 years) were evaluated. The FMS and EF were evaluated using the Test of Gross Motor Development-2 (TGMD-2) and the NIH Toolbox Cognition Battery (NTCB), respectively.

**Results:**

Total FMS score was moderately and positively correlated with total EF score (*r* = 0.33, *p* < 0.001) and was a significant predictor of total EF score (β = 0.37, *p* < 0.001). Specifically, locomotor skills were significant predictors of inhibition control (β = 0.21, *p* < 0.001), working memory (β = 0.18, *p* < 0.01), and cognitive flexibility (β = 0.24, *p* < 0.001), while object control skills were only significant predictors of inhibition control (β = 0.17, *p* < 0.01).

**Conclusion:**

FMS were significantly and positively correlated with EF and were significant predictors of EF. Early childhood policymakers, preschool teachers, and researchers should take these connections seriously and implement appropriate complex motor intervention programs in future teaching to stimulate the development of both motor and higher-order cognitive skills in preschool children.

## Introduction

In the past years, researchers have paid more attention to the relationship between motor skills and executive function (EF) in young children due to the close link between brain development and motor skills (Diamond, 2000). There are some preliminary theoretical explanations for the interrelations between motor skills and EF. According to the embodied cognition theory, cognition, including EF, is based on motor development (Foglia and Wilson, 2013). Motor development offers new opportunities for young children to actively explore their physical and social environment through a perception-action cycle, which promotes cognitive development (von Hofsten, 2007). The acquisition of new cognitive abilities in turn allows children to develop more complicated motor skills (Adolph and Hoch, 2019). In addition, the theory of reciprocity and automaticity have been proposed to help understand the positive interaction between motor skills and EF. Reciprocity theory holds that motor skills and cognitive skills develop mutually through interaction with the environment, namely that motor experience fosters the acquisition of motor abilities and then promotes interaction with the environment, which ultimately facilitates the formation of higher cognitive processes (Campos et al., 2000; McClelland and Cameron, 2018). Automaticity theory suggests that the completion of motor and cognitive tasks will compete for the same limited cognitive attention resources. The completion of an initial motor task demands the allocation of cognitive-attention resources, but since repeated practice leads to automatic behavior, the cognitive attention resources required for successful performance will be reduced (Floyer-Lea and Matthews, 2004). Therefore, if a certain motor skill is automated, more attention resources will be used to perform cognitive processes (Cameron et al., 2015). Accordingly, EF will no longer be involved in automated motor tasks, making it easier to perform another EF-demanding task simultaneously (Floyer-Lea and Matthews, 2004).

When reviewing the empirical studies on the association between motor skills and EF in preschool children, it was discovered that these studies used different and sometimes limited sets of motor skills and/or EF, which led to it being very difficult to compare the findings across studies (Livesey et al., 2006; Lehmann et al., 2014; Houwen et al., 2017; Oberer et al., 2017; Maurer and Roebers, 2019; Van der Veer et al., 2020). And thus, the relationship between them has not been clearly understood so far. As a result, it is critical to further explore whether and to what extent the relationship between motor skills and EF exists at the item level, since this evidence may have some implications for the design of early intervention (Van der Veer et al., 2020).

Early childhood is a crucial stage for the development of fundamental motor skills (FMS) (Gallahue et al., 2011). Many governments have prioritized efforts to promote FMS for young children, owing to the fact that these initiatives provide a variety of life-long benefits (i.e., enhancing physical fitness, increasing physical activity, reducing sedentary behaviour, improving weight status, etc.) (Lubans et al., 2010; Jones et al., 2011; Morrow et al., 2013). Additionally, some studies have found a positive correlation between FMS in early childhood and subsequent health, cognitive ability, self-confidence (Lubans et al., 2010; Robinson et al., 2015; Schmidt et al., 2017).

Early childhood is not only a critical period for the development of FMS but also for the development of EF. EF refers to the cognitive processes that are required for the conscious, top-down control of action, thought, and emotions and that are associated with neural systems involving the prefrontal cortex (Lerner et al., 2015). There is general agreement that EF includes three core cognitive competences: inhibition control (resisting temptations and not acting impulsively), working memory (storing information in mind and using it creatively), and cognitive flexibility (shifting thoughts or strategies for a problem to adapt flexibly to new demands, rules, or priorities) (Miyake et al., 2000; Diamond, 2013). EF, as an advanced cognitive function, plays an important role in lifelong development and develops rapidly throughout the preschool years (Anderson, 2002; Best and Miller, 2010). There is evidence to show that the EF of preschoolers can significantly predict academic achievement in childhood and provide advantages for children’s school readiness (Blair and Razza, 2007; Oberer et al., 2018). In addition, young children’s EF is negatively correlated with negative lifestyle behaviors (e.g., impulsiveness, aggressive behavior) in late growth (Hughes and Ensor, 2008; Moffitt et al., 2011; Utendale and Hastings, 2011) and positively correlated with interpersonal communication, health, wealth, and quality of life (Banich, 2009; Moffitt et al., 2011; Houwen et al., 2017; Caporaso et al., 2019; Huang et al., 2020).

As far as we know, there are a few studies that have focused on the association between FMS and EF in preschool children (Maurer and Roebers, 2019). For example, Oberer et al. found that there was a significant positive correlation between preschoolers’ FMS and EF, but this study only measured a select number of fundamental locomotor skills (e.g., one-leg-stand, jumping sideways, moving sideways), and did not involve object control skills (Oberer et al., 2017). Cook et al. found that there was a partial correlation between preschoolers’ FMS and EF, and FMS was positively correlated with inhibition control and working memory but not with cognitive flexibility (Cook et al., 2019). In addition, these studies used different testing tools for EF and FMS, such as Oberer et al. used product-oriented motor tests, while Cook et al. used process-oriented motor tests.

However, no study has focused on the association between FMS and EF in preschool children in China. Children are naturally engaged more in FMS in their preschool years, and thus, further exploration of the relationship between FMS and EF in young children is desirable and necessary due to the lack of consistent and convincing evidence (Diamond and Lee, 2011; Oberer et al., 2017). As a result, we used international authoritative measurement tools, the Test of Gross Motor Development-2 (TGMD-2) and the NIH Toolbox Cognition Battery (NTCB), to assess children’s FMS and EF in this study, respectively. The main purpose of this study was to explore the association between early FMS and EF in a sample of preschool children in China. Specifically, the study aimed to examine whether and how components of FMS are correlated with components of EF in Chinese preschool children. This can not only accumulate evidence for a better understanding of the relationship between FMS and EF, but also help to guide the design of interventions to promote early childhood development.

## Materials and methods

### Participants

The participants were recruited through geographical proximity, convenience, and cluster random sampling from four urban and two rural preschools in Shanxi (China).

A total of 473 young children were invited to participate in the study. The inclusion criteria for participating in the study were as follows: (1) submit signed informed consent agreeing to engage in the study signed by their parents or legal guardians; (2) not have an illness or disability (physical or mental) and be able to participate in physical activities normally; (3) subjects’ age between 3 and 5 years; (4) complete all measurements. As a result, 79 children who did not meet the inclusion criteria were excluded. Finally, 394 children were considered in the present study. All children could withdraw from the study at any time if they felt uncomfortable during the test or for other reasons. The study protocol was approved by the Ethics Committee of Capital University of Physical Education and Sports (code 2021A28), abiding by the Helsinki Declaration amended in Fortaleza (Brazil) in 2013.

### Measurements

Before starting the test, the research group contacted the person in charge of the kindergarten to explain the purpose of this study. Following permission, the necessary sociodemographic data (i.e., gender, birthdate, and handedness) was gathered, and the measurements were conducted in a kindergarten setting by the trained tester who used standardized equipment and followed the same process according to the examiner’s instructions. The trained testers were made up of nine graduate students in Pedagogical and Educational Sciences and Human Movement Sciences, two of whom were responsible for testing locomotor skills and object control skills, respectively. Before they were allowed to administer the tests, they had to follow and pass an extensive training. As part of the training, they were first asked to read the manual and watch the video on their own, and attended two online workshops on interpreting the manual. And then, they followed three offline training sessions, which mainly included site layout, test explanation, action demonstration, information input, data collection, instrument use, and so on. Additionally, five children were invited to participate in a pre-test in a training session. Finally, these testers rehearsed the whole process of testing, mastered the testing methods, and unified the testing standards.

#### Anthropometry

A stadiometer (SECA 213, Hamburg, Germany) and a balancing scale (MIUI 2, Anhui, China) were used to measure height (cm) and weight (kg). Body mass index (BMI) was computed by dividing weight in kilograms by height in meters squared.

#### Fundamental motor skills

The FMS were evaluated using the TGMD-2, which has been validated and standardized as a widely used measure of FMS for young children (Ulrich, 2000). The TGMD-2 has been demonstrated to be suitable for the Chinese context (Li and Ma, 2007). The TGMD-2 consists of two subtests, namely locomotor skills (i.e., run, gallop, hop, leap, horizontal jump, and slide) and object control skills (i.e., striking a stationary ball, stationary dribble, catch, kick, overhand throw, and underhand roll). Each skill has 3–5 performance criteria. These test tasks were administered by the trained examiner on the kindergarten’s outdoor playground. The entire TGMD-2 took approximately 15–20 min for one child. The subjects were separated into groups of 5–7 for the assessment. A 3-min warm-up that comprised running and jumping games was conducted before the testing. It should be noted that each child had a practice trial before performing each of these test tasks on the TGMD-2 in which the examiner could correct any errors. However, no instructions were offered during the testing. The performance of all subjects was recorded on video and scored by the trained researchers. These researchers had previously established high inter-rater reliability for 12 skills, ranging from 0.90 (striking a stationary ball) to 0.97 (horizontal jump) and high intra-rater reliability, ranging from 0.91 (stationary dribble) to 0.97 (run), which have been published elsewhere (Zhao et al., 2022). The score of each subtest was calculated by adding up the scores of the six skills it contains, and the total FMS score was calculated by adding up the scores of both subtests. More information on TGMD-2 may be found elsewhere (Ulrich, 2000).

#### Executive function

The EF was evaluated using the NTCB, among which there are three tasks suitable for testing children’s EF, namely the NIH Toolbox Flanker Inhibitory Control and Attention Test Ages 3–7 for measuring inhibition control; the NIH Toolbox List Sorting Working Memory Test Ages 3–6 for measuring working memory; and the NIH Toolbox Dimensional Change Card Sort Test Ages 3–7 for measuring cognitive flexibility, which have also been validated in children aged 3–6 years (Bauer and Zelazo, 2013; Weintraub et al., 2013), and all data processing can be fully automated. These test tasks were performed by the trained examiner using an iPad application in a relatively quiet classroom or conference room and took 8–10 min for each task. Additionally, considering that it would take a long time to perform the NTCB and that acute strenuous exercise could affect the accuracy of the NTCB, the NTCB was arranged to be carried out on the second day after the TGMD was completed. The total EF score was calculated by adding up the scores of the three tasks. More detailed descriptions of each measure can be found in previous studies (Weintraub et al., 2013; Akshoomoff et al., 2014), or can be accessed directly from the official website.^1^

### Statistical analysis

The statistical analyses were performed using the SPSS software (SPSS v.24, IBM Corporation, New York, United States), with a statistical significance set at *p* < 0.05. The characteristics of the participants were summarized using descriptive statistics for continuous (means and standard deviation, *M* ± *SD*) and categorical (percentage, %) data. In the preliminary analysis, there were three outliers in the data for object control skills, as assessed by the pre-specified criteria, namely that the values beyond *M* ± 3*SD* of outcome variables were considered as outliers. Since the inclusion of these outliers had no significant impact on the study results, we chose to retain them in the final analysis. The normality of outcome variables was assessed using the Kolmogorov-Smirnov test, histograms, and normal quantile–quantile (Q-Q) plots. All these variables analyzed in the study did not show significant deviations from the Gaussian distribution. Partial correlation analysis was used to analyze the correlation between the components of FMS and EF, after controlling the age, gender, BMI, and setting. The partial correlation coefficient was considered small (0–0.30), moderate (0.31–0.49), large (0.50– 0.69), very large (0.70–0.89), and almost perfect (0.90–1.00) (Cavedon et al., 2022). In addition, the multiple linear regression models estimated by ordinary least squares (OLS) were used to assess the relative contribution of the FMS as predictors of EF and the specific contribution of the components of FMS to the components of EF (Cook et al., 2019), while controlling for age, sex, BMI and setting. The multicollinearity between independent variables was determined using the variance inflation factor (VIF). The VIF < 5 suggested that multicollinearity was not a problem in the models.

## Results

A total of 394 healthy preschool children (4.07 ± 0.76 years) were included in the final analysis, of which 182 (46.2%) were girls and 212 (53.8%) were boys. The demographic characteristics of participants and descriptive statistics of measured variables are summarized in Table 1.

**TABLE 1:** Descriptive statistics of study variables.

Variables	*M* ± *SD*/%
**Demographics**	
Age (years)	4.07 ± 0.76
Sex (girls)	46.20%
Setting (urban)	67.30%
BMI (kg/m^2^)	15.36 ± 1.48
**Fundamental motor skills**	
Total	34.29 ± 9.06
Locomotor	17.44 ± 5.54
Object control	16.85 ± 4.58
**Executive function**	
Total	177.76 ± 42.80
Inhibition control	55.74 ± 21.57
Working memory	61.23 ± 13.62
Cognitive flexibility	60.78 ± 16.89

Results were expressed as means and standard deviation (M ± SD) for continuous variables and percentage (%) for categorical variables. BMI, body mass index.

The result of partial correlation analyses (Table 2) indicated that total FMS score was moderately and positively correlated with total EF score (*r* = 0.33, *p* < 0.001). Specifically, locomotor skills and object control skills were significantly positively correlated with all three components of EF (*r* from 0.12 to 0.25, *p* < 0.05), respectively, with a higher partial correlation coefficient in locomotor skills.

**TABLE 2:** Partial correlation outcomes of measured variables.

Variables	1	2	3	4	5	6	7
1. Locomotor	–						
2. Object control	0.35*	–					
3. Total FMS	0.88*	0.76*	–				
4. Inhibition control	0.26*	0.22*	0.29*	–			
5. Working memory	0.18*	0.12^$^	0.19*	0.28*	–		
6. Cognitive flexibility	0.23*	0.15^#^	0.24*	0.42*	0.25*	–	
7. Total EF	0.31*	0.22*	0.33*	0.82*	0.62*	0.77*	–

Partial correlation was conducted by adjusting for age, sex, BMI, and setting. FMS, fundamental motor skills; EF, executive function.

*p < 0.001; ^#^p < 0.01; ^$^p < 0.05.

The multiple linear regression analysis outcomes (Table 3) demonstrated that total FMS was a significant predictor of total EF (Adj.*R*^2^ = 0.47, β = 0.37, *p* < 0.001). Furthermore, the specific influence of the locomotor and object control skills on the components of EF was evaluated. In terms of inhibition control, the regression model revealed that locomotor skills and object control skills were significant predictors (Adj.*R*^2^ = 0.41, *p* < 0.01), and locomotor skills (β = 0.21) had a greater impact on inhibition control compared to object control skills (β = 0.17). With respect to working memory, the regression model revealed that locomotor skills were a significant predictor (Adj.*R*^2^ = 0.29, β = 0.18, *p* < 0.01), while object control skills were not (*p* = 0.26). Additionally, the regression model discovered that locomotor skills were also a significant predictor of cognitive flexibility (Adj.*R*^2^ = 0.25, β = 0.24, *p* < 0.001), but object control skills were not (*p* = 0.14).

**TABLE 3:** The multiple linear regression analysis outcomes of FMS predicting performance on EF.

EF	Predictors	*B*	*SE B*	β	*t*	*p*	*R* ^2^	Adj. *R*^2^
Total EF								
Model 1	Age	34.831	2.195	0.621	15.870	<0.001*	0.417	0.411
	Sex *^a^*	−11.582	3.344	−0.135	−3.464	0.001^#^		
	BMI	−0.421	1.127	−0.015	−0.374	0.709		
	Setting*^b^*	4.598	3.563	0.050	1.291	0.198		
Model 2	Age	20.232	2.972	0.361	6.807	<0.001*	0.481	0.474
	Sex*^a^*	−12.378	3.164	−0.144	−3.912	<0.001*		
	BMI	−0.187	1.066	−0.006	−0.175	0.861		
	Setting*^b^*	−1.212	3.474	−0.013	−0.349	0.727		
	Total FMS	1.763	0.257	0.373	6.862	<0.001*		
Inhibition control								
Model 1	Age	16.620	1.154	0.588	14.408	<0.001*	0.367	0.360
	Sex*^a^*	−4.305	1.758	−0.100	−2.450	0.015^$^		
	BMI	0.411	0.592	0.028	0.694	0.488		
	Setting*^b^*	1.611	1.873	0.035	0.860	0.390		
Model 2	Age	9.925	1.637	0.351	6.063	<0.001*	0.419	0.410
	Sex*^a^*	−4.615	1.793	−0.107	−2.574	0.010^$^		
	BMI	0.520	0.569	0.036	0.914	0.361		
	Setting*^b^*	−1.092	1.869	−0.024	−0.584	0.559		
	Locomotor	0.829	0.210	0.213	3.945	<0.001*		
	Object Control	0.789	0.286	0.168	2.759	0.006^#^		
Working memory								
Model 1	Age	8.925	0.780	0.500	11.439	<0.001*	0.273	0.265
	Sex*^a^*	−1.516	1.189	−0.056	−1.275	0.203		
	BMI	−0.209	0.401	−0.023	−0.521	0.603		
	Setting*^b^*	2.504	1.267	0.086	1.977	0.049^$^		
Model 2	Age	6.161	1.135	0.345	5.429	<0.001*	0.300	0.289
	Sex*^a^*	−1.354	1.243	−0.050	−1.089	0.277		
	BMI	−0.154	0.394	−0.017	−0.391	0.696		
	Setting*^b^*	1.190	1.295	0.041	0.919	0.359		
	Locomotor	0.446	0.146	0.181	3.060	0.002^#^		
	Object Control	0.226	0.198	0.076	1.137	0.256		
Cognitive flexibility								
Model 1	Age	9.286	1.007	0.420	9.221	<0.001*	0.212	0.204
	Sex*^a^*	−5.761	1.534	−0.170	−3.755	<0.001*		
	BMI	−0.623	0.517	−0.055	−1.205	0.229		
	Setting*^b^*	0.483	1.635	0.013	0.296	0.768		
Model 2	Age	4.754	1.447	0.215	3.285	0.001^#^	0.259	0.248
	Sex*^a^*	−5.505	1.585	−0.163	−3.472	0.001^#^		
	BMI	−0.534	0.503	−0.047	−1.061	0.289		
	Setting*^b^*	−1.665	1.652	−0.046	−1.007	0.314		
	Locomotor	0.727	0.186	0.238	3.916	<0.001*		
	Object Control	0.373	0.253	0.101	1.474	0.141		

FMS, fundamental motor skills; EF, executive function; BMI, body mass index.

^a^0 = girls, 1 = boys; ^b^0 = rural, 1 = urban.

*p < 0.001; ^#^p < 0.01; ^$^p < 0.05.

## Discussion

The main objective of the current study was to explore the relationships between early FMS and EF in a sample of preschool children in China. Specifically, this study aimed to examine whether and how components of FMS are correlated with components of EF in Chinese preschool children.

The findings acquired in this study indicate that total FMS score is significantly and positively correlated with total EF score and is a significant predictor of total EF score. Moreover, locomotor skills and object control skills were significantly positively correlated with all three components of EF, respectively. These results are partly in line with what has been demonstrated in previous studies (Oberer et al., 2017; Cook et al., 2019). For instance, Oberer et al. found that there was a significant positive correlation between FMS and EF, but this study only measured a select number of fundamental locomotor skills and did not involve object control skills (Oberer et al., 2017). In addition, Cook et al. found that there was a correlation between FMS and EF, and FMS was positively correlated with inhibitory control and working memory but not with cognitive flexibility (Cook et al., 2019). Therefore, in view of the differences in measured variables and measured tools in previous studies, the findings of these studies should be compared with caution to the current study. Evidence acquired from neuroimaging studies brings some interpretation for this association. Studies have shown that the cerebellum and prefrontal region will be synergistically activated when completing complex and novel tasks that require quick response and concentration, given that the coordination of complex movements requires the participation of EF and is also controlled by the cerebellum (Diamond, 2000; Tomporowski et al., 2015). FMS consist of a number of movements that coordinate the body’s large muscles to maintain balance and effectively move the trunk and limbs, and they are related to the formation of neuronal myelin sheath in the brain area controlling balance and coordination (Feldman, 2017). FMS provides new opportunities for young children to actively explore their physical and social environment through a perception-motion cycle, which promotes cognitive development (von Hofsten, 2007), and the acquisition of new cognitive abilities in turn allows children to develop more complicated motor skills (Adolph and Hoch, 2019). In addition, studies have demonstrated that more advanced FMS are associated with higher levels of physical activity and higher levels of physical fitness (Stodden et al., 2008; Robinson et al., 2015). According to the neurotrophic hypothesis, higher levels of physical activity in turn increase metabolic activity and trigger a cascade of biochemical changes (e.g., promoting the secretion of brain-derived neurotrophic factors) that can enhance brain plasticity and change brain structure and function (Lippi et al., 2020). This is also a possible explanation for the above association found in this study.

Furthermore, with respect to the specific association of components of FMS and EF, the results of the present study demonstrate that locomotor skills are significant predictors of all three components of EF, while object control skills are only significant predictors of inhibition control. This finding is partly consistent with those found in the previous study, showing locomotor skills are significant predictors of inhibition control and working memory, whereas object control skills are significant predictors of inhibition control (Cook et al., 2019). However, due to the use of different EF testing tools, it should be interpreted with caution. Generally speaking, inhibition control would be critical to maintain attention and avoid distraction during non-automated physical activity. Yet locomotor skills appeared to be specifically related to working memory and cognitive flexibility, which may be due to the fact that locomotor tasks take longer to accomplish than discrete tasks, thus forcing the infant to keep the locomotor goal in mind for a longer period of time (Anderson et al., 2013), and locomotor skills place greater demands on memory, activation, and switching of movement sequences (Alesi et al., 2016), such as hopping and sliding, which not only require additional coordination demands but also require children to exchange support legs and shift the direction of movement.

Besides that, it should be noted that more attention is paid to physical fitness than to motor development for preschool children in China. China’s official physical fitness test indicators for young children include eight indicators (General Administration of Sport of China, 2003), of which four (e.g., 2 × 10 m shuttle run test, standing long jump test, continuous jump on both feet test, and balance beam walk test) are related to locomotor skills, which will make children’s locomotor skills develop better. However, object control skills are more difficult because they not only require the coordination of upper-and lower-limbs and trunk but also require hands or feet to manipulate equipment, and the current kindergartens do not pay attention to the teaching of object control skills, which leads to the overall low level of children’s object manipulation skills, and finally leads to the lower statistical power associated with EF. This may also be one reason why there is a different correlation between locomotor skills, object control skills, and components of EF. Further studies are needed to identify the conformance, direction, and potential mechanisms of these links.

As a result, these findings can be of significant practical implications in the educational setting, particularly for the construction of preschool curricula and the selection of the most effective physical activities taken to promote the development of preschool children’s EF. Early childhood policy makers, kindergarten teachers, and researchers should seriously consider these findings and attach importance to the teaching of FMS for preschool children. According to our existing research, the exercise of locomotor skills may be an important means of physical exercise to promote the development of preschool children’s EF. When designing the exercise program, it can be considered to integrate the requirements of cognitive conditions (e.g., making opposite or the same actions according to different signals; helping and guiding children to remember the key steps of different tasks; and adjusting their behavior according to different task requirements, etc.), which can pose a challenge to the development of children’s EF. However, considering the importance of FMS to children’s development, the practice of object control skills should also be taken seriously. This can provide more possibilities for the improvement of children’s physical activity, learning ability, and cognitive level.

## Strengths and limitations

Strength of our study is that it is the first to explore the association between FMS and EF in preschool children in China. What’s more, the FMS and EF were measured using TGMD-2 and NTCB, respectively, which are international authoritative test tools, improving the validity and reliability of the results and allowing insight into the specific association of components of FMS and EF. Besides that, the sample size of this study is larger than that of many previous studies, which also contributes to the dependability of the results.

Regarding the limitations of the present study, our findings must be interpreted within specific settings, and the sample may not be representative of China because the sample size is small and the distribution of selected kindergartens is not random. Additionally, we did not consider physical activity, family parameters (e.g., family income), and kindergarten parameters, which may influence FMS and EF. Finally, the cross-sectional study design limited inferences about the direction of these relationships to some extent.

## Conclusions

It is concluded that FMS are significantly and positively correlated with EF and are significant predictors of EF. Specifically, locomotor skills are associated with all three components of EF, while object control skills are only associated with inhibition control. Early childhood policymakers, preschool teachers, and researchers should take these connections seriously and implement appropriate complex motor intervention programs in future teaching to stimulate the development of both motor and higher-order cognitive skills in preschool children.

## Data availability statement

The raw data supporting the conclusions of this article will be made available by the authors, without undue reservation.

## Ethics statement

The studies involving human participants were reviewed and approved by the Ethics Committee of Capital University of Physical Education and Sports (code 2021A28), abiding by the Helsinki Declaration Amended in Fortaleza (Brazil) in 2013. Written informed consent to participate in this study was provided by the participants’ legal guardian/next of kin.

## Author contributions

XH and JX: conceptualization. XH: software. MZ and XH: formal analysis and visualization. MZ, ZK, and XH: investigation. XH, MZ, and JX: writing—review and editing. All authors have read and agreed to the published version of the manuscript.

## Footnotes

1
https://www.healthmeasures.net/explore-measurement-systems/nih-toolbox/intro-to-nih-toolbox
